# *FUS* and *TARDBP* but Not *SOD1* Interact in Genetic Models of Amyotrophic Lateral Sclerosis

**DOI:** 10.1371/journal.pgen.1002214

**Published:** 2011-08-04

**Authors:** Edor Kabashi, Valérie Bercier, Alexandra Lissouba, Meijiang Liao, Edna Brustein, Guy A. Rouleau, Pierre Drapeau

**Affiliations:** 1 Department of Pathology and Cell Biology and Groupe de Recherche Sur le Système Nerveux Central, University of Montreal, Montreal, Canada; 2 Center of Excellence in Neuromics, Centre Hospitalier de l'Universite de Montreal, Montreal, Canada; 3 Department of Medicine, University of Montreal, Montreal, Canada; University of Minnesota, United States of America

## Abstract

Mutations in the *SOD1* and *TARDBP* genes have been commonly identified in Amyotrophic Lateral Sclerosis (ALS). Recently, mutations in the Fused in sarcoma gene (*FUS*) were identified in familial (FALS) ALS cases and sporadic (SALS) patients. Similarly to TDP-43 (coded by *TARDBP* gene), FUS is an RNA binding protein. Using the zebrafish (*Danio rerio*), we examined the consequences of expressing human wild-type (WT) *FUS* and three ALS–related mutations, as well as their interactions with *TARDBP* and *SOD1*. Knockdown of zebrafish *Fus* yielded a motor phenotype that could be rescued upon co-expression of wild-type human *FUS*. In contrast, the two most frequent ALS–related *FUS* mutations, R521H and R521C, unlike S57Δ, failed to rescue the knockdown phenotype, indicating loss of function. The R521H mutation caused a toxic gain of function when expressed alone, similar to the phenotype observed upon knockdown of zebrafish *Fus*. This phenotype was not aggravated by co-expression of both mutant human *TARDBP* (G348C) and *FUS* (R521H) or by knockdown of both zebrafish *Tardbp* and *Fus*, consistent with a common pathogenic mechanism. We also observed that WT *FUS* rescued the *Tardbp* knockdown phenotype, but not *vice versa*, suggesting that *TARDBP* acts upstream of *FUS* in this pathway. In addition we observed that WT *SOD1* failed to rescue the phenotype observed upon overexpression of mutant *TARDBP* or *FUS* or upon knockdown of *Tardbp* or *Fus*; similarly, WT *TARDBP* or *FUS* also failed to rescue the phenotype induced by mutant *SOD1* (G93A). Finally, overexpression of mutant *SOD1* exacerbated the motor phenotype caused by overexpression of mutant *FUS*. Together our results indicate that *TARDBP* and *FUS* act in a pathogenic pathway that is independent of *SOD1*.

## Author Summary

Mutations in the *SOD1*, *TARDBP*, and *FUS* genes have been commonly identified in Amyotrophic Lateral Sclerosis (ALS). However, possible interactions between these ALS–causative genetic mutations have not been examined. Here we expressed each of three human *FUS* mutations (R521H, R521C, and S57Δ) in zebrafish embryos, with or without knocking down the zebrafish homolog *Fus*, and observed a motor phenotype consisting of significant behavioral (touch-evoked escape response) and cellular (shortened axonal projections from motor neurons) deficits due to loss of function for the R521H and R521C mutations and/or toxic gain of function solely for the R521H mutation. Wild-type *FUS* could rescue the *Tardbp* knockdown phenotype, but not *vice versa*, suggesting that *TARDBP* is upstream of *FUS* in this pathway responsible for motor neuron disorder. Furthermore, neither *TARDBP* nor *FUS* were able to modify and/or rescue the motor phenotype caused by mutant *SOD1*, and likewise *SOD1* failed to rescue the phenotype of zebrafish expressing mutant *TARDBP* or *FUS*. Our results indicate that *TARDBP* acts upstream of *FUS* in a pathogenic pathway that is distinct from that of *SOD1*.

## Introduction

ALS is the most common motor neuron disorder and is characterized by loss of upper and lower motor neurons. It is the third most common neurological disorder with an incidence of 1–2 people in 100,000, a prevalence of 4–6 per 100,000 and with a lifetime risk of 1 in 1,000 [1], [2]. ALS has a devastating course with disease onset generally first detected at 50–60 years of age followed by rapid muscle weakness, atrophy and eventual paralysis resulting in death due to respiratory failure within 1–5 years. Approximately 10% of ALS patients have a familial history for this disease (FALS), whereas the majority (90%) of cases appears to be of a sporadic nature (SALS) [3], [4]. So far, three major genes have been implicated in ALS: *SOD1*, *TARDBP* and *FUS*. However, it is not known whether these three genes interact in a common pathway or represent distinct ALS etiologies.

Cu/Zn superoxide dismutase (*SOD1*) mutations were the first to be identified predominantly in FALS patients in 1993, with more than 130 mutations currently identified in approximately 20% of FALS patients [2], [4]–[6]. A toxic gain of function of mutant SOD1 causes pronounced motor deficits *in vivo* correlated to motor neuron degeneration in a number of animal models [2], [7], [8]. Recently, mutations in TAR DNA binding protein (*TARDBP*) [9]–[12] and Fused in sarcoma (*FUS*) [13], [14] genes were found in both FALS and SALS patients, opening novel possibilities of studying the predominant, sporadic form of this disease [11], [15]. In 2008, two concurrent reports, including ours, identified a dozen missense mutations in the *TARDBP* gene [9]–[11]. So far, 38 *TARDBP* mutations have been identified predominantly clustered in the C-terminus, glycine-rich region of the TDP-43 protein encoded by the *TARDBP* gene in approximately1% of SALS and 3% of FALS [11], [16]. Two concurrent publications in 2009 identified *FUS* mutations that occur in about 5% of FALS and less than 1% of SALS [11], [16] with 35 mutations so far identified in ALS cases. Similarly to TDP-43 (encoded by *TARDBP*), FUS is an RNA binding protein mainly localized in the nucleus [11]. Further comparable to the *TARDBP* mutations identified in ALS cases, most of the *FUS* mutations cluster in the C-terminus of the FUS protein, including the most common mutations, R521C present in 22 FALS and 4 SALS and R521H present in 9 FALS and 4 SALS [11], [13], [14], [17]–[28]. Interestingly, both proteins have been found to be major components of ubiquitinated inclusion bodies in autopsy tissue, not only in ALS patients, but in a number of commonly-related neurodegenerative disorders, such as frontotemporal lobar degeneration (FTLD), Alzheimer's and Parkinson's diseases [11], [16]. In rare cases, either *TARDBP* or *FUS* mutations have also been identified in FTLD cases with or without motor neuron involvement [29]–[32].

In line with this genetic evidence, pathological reports have shown that TDP-43 and FUS antibodies co-label protein aggregates consisting of inclusion bodies observed in both SALS and FALS cases [33], distinguishing these aggregates from the SOD1 positive found in FALS patients [34]. Two recent studies in cell lines have shown that TDP-43 and FUS interact [35] with a report showing that both proteins are able to influence *HDAC6* mRNA production [36]. Strong overexpression of both *TARDBP* and *FUS* (either WT or carrying ALS mutations) were shown to exacerbate a degenerative phenotype in the *Drosophila* eye as compared to either gene alone [37]. Further, recent studies have also demonstrated that depletion of *TARDBP* using RNAi in cell lines or *tardbp* in *Drosophila* leads to specific decrease in *FUS* mRNA levels [38], [39]. Overall, this evidence suggests that these proteins are involved in similar pathogenic mechanisms leading to motor neuron degeneration. However, the relevance of *TARDBP* and *FUS* mutations to these pathogenic mechanisms is not well understood, since none of the known functions of TDP-43 and FUS, such as binding to RNA, DNA and heterogeneous nuclear ribonucleoproteins (hnRNPs), have been shown to be perturbed by ALS-related mutations [36], [40], [41].

Recent insight in neurodegenerative diseases has revealed that several genes mutated in these disorders could participate in common molecular mechanisms, raising the possibility of a multigenic interaction at the root of the pathogenesis of neurodegeneration. One such example is the well-established genetic and functional interaction between PTEN-induced putative (mitochondrial) kinase 1 (*PINK1*) and the E3 ubiquitin ligase parkin (*PRKN*) [42], [43], two genes mutated in Parkinson's disease [44], [45]. Another recent example is the identification of interactions between TDP-43 and ataxin-2 *in vivo*, with ataxin-2 containing polyglutamine expansions being a potent modifier of TDP-43 toxicity [46]. This interaction was also found to have pathogenic implications since SCA1 triplet repeats are significantly increased in ALS patients as compared to controls [46].

In order to establish whether genetic interactions also exist in ALS pathogenesis we carried out a multigenic analysis of *FUS*, *TARDBP* and *SOD1* in zebrafish. Zebrafish are proving to be a valuable vertebrate model to further our understanding of these multigenic interactions [47]. A number of recent studies in zebrafish with relevance to motor neuron diseases [8], [48]–[52] suggest that this vertebrate organism is ideally suited as a model to rapidly and efficiently replicate certain aspects of these disorders [53]. This allows us to better understand genetic (and eventually the cellular and molecular) mechanisms of motor neuron degeneration during a much shorter time span (in this case inside a few days). Further, these models allow us to define the crucial steps in disease development and to find ways to interfere with the development of early hallmarks of the disease, rather than to exactly replicate the (likely later) symptoms.

We have previously demonstrated that mutations of *TARDBP* cause a pronounced motor phenotype characterized by aberrant motor neuron morphology and motor behavior in zebrafish through both toxic gain and loss of function [50], with the toxic gain of function results being recently confirmed by another group [48]. Up to now, this represents the only vertebrate model of mutant *TARDBP* where a motor neuron disorder has been observed when compared to similar expression of WT *TARDBP*. Here we report that *FUS* mutations identified in ALS patients present a similar motor phenotype. Our results reveal a common genetic pathway for *FUS* and *TARDBP in vivo*, with *FUS* possibly acting downstream of *TARDBP*. These results also establish that *SOD1* acts independently of *FUS/TARDBP* in causing a motor phenotype in these genetic models of ALS. The establishment of these new genetic models for ALS provides new tools to study the molecular mechanisms of neurodegeneration.

## Results

### FUS mutations characterized in this study

Three ALS-related mutations identified in our patient cohort at the University of Montréal Health Centre (CHUM; individuals from Quebec and France) were selected for this study. The R521C is the most common *FUS* mutation identified so far in 22 FALS and 4 SALS in a number of cohorts. The R521H mutation is also a very common mutation accounting for 9 FALS and 4 SALS [11], [13], [14], [17]–[28]. Both of these mutations have been shown to mislocalize from the nucleus to the cytosol in a number of cell line experiments [14], [54], [55]. The S57Δ mutation was identified in one SALS patient from our cohort and is one of the only variants identified in ALS cases to be located in the N-terminus region of the FUS protein [19].

### Loss of function of *Fus* leads to a motor phenotype that can be rescued by WT *FUS* but not ALS–related mutations

**Figure 1:**
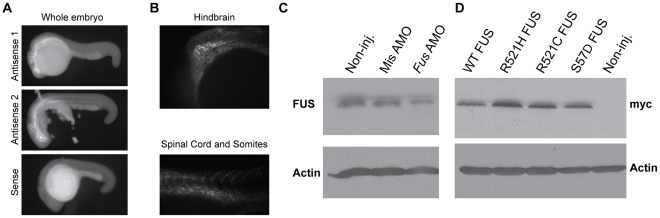
*Fus* mRNA expression in early development of zebrafish and Fus levels are reduced upon KD with a specific AMO. A) *In situ* hybridization with antisense (upper panels) and sense (lower panel) oligo probes specific to *Fus* mRNA revealed a predominant expression in the CNS, in particular in the hindbrain, spinal cord, as well as intersomitic segments as shown in B) in 24 hpf zebrafish embryos. C) Western blot analysis of zebrafish embryos injected with an AMO that specifically binds and inhibits *Fus* mRNA translation, showing reduced levels of Fus expression as compared to non-injected zebrafish embryos and zebrafish embryos injected with an AMO, where 5 nucleotides are mismatched (mismatch). D) WT human *FUS* mRNA or one of three ALS-related *FUS* mutations, each tagged with myc, were overexpressed in zebrafish and larval extracts were collected. Immunoblotting with myc antibody reveals similar expression in all these extracts.

We first confirmed by *in situ* hybridization that *Fus* mRNA was indeed expressed as early as 24 hours post-fertilization (hpf) in zebrafish embryos (Figure 1A) mainly in the hindbrain, eye and intersomitic segments as well as the spinal cord (Figure 1B). The antisense morpholino oligonucleotide (AMO) for *Fus* KD was designed to specifically bind near the ATG of zebrafish *Fus* but nowhere else in the zebrafish genome. As a control, a mismatch AMO was also designed that does not bind anywhere in the zebrafish genome. Western blot analysis using a FUS antibody demonstrated knockdown (KD) of Fus expression by approximately 60% solely in fish injected with an AMO designed to bind *Fus*, but not to the mismatch AMO control (Figure 1C).

**Figure 2:**
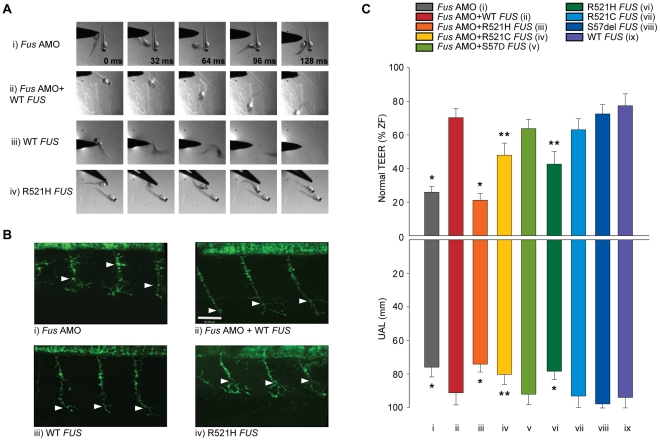
ALS-related *FUS* mutations cause a motor phenotype through both gain and loss of function. A) KD of *Fus* (i) causes a major deficit in the TEER, which can be rescued by WT *FUS* mRNA (ii). A similar motor phenotype is also observed upon overexpression of mutant R521H *FUS* mRNA (iv), but not WT *FUS* mRNA alone (iii). B) Immunocytochemical analysis of axonal projections from spinal cord motor neurons revealed a marked reduction of primary axonal length (arrowheads represent the unbranched axonal length; UAL) upon KD of zebrafish *Fus*, which could be rescued by co-expression of WT *FUS* mRNA (ii). A similar axonal phenotype is also observed upon overexpression of mutant *FUS* mRNA (iv), but not present in zebrafish expressing WT *FUS* mRNA (iii) alone. C) Both the percentage of fish with normal TEER (upward bars, averages of % of normal zebrafish (ZF) embryos ± standard errors of mean, SEM) and the length of primary motor axons (downward bars, averages of UAL in µm ± SEM) were unaffected or significantly reduced (* for p<0.05 from WT *FUS* mRNA injections; ** for p<0.05 from WT *FUS* mRNA injections and *Fus* AMO; all values given in Table 1) when compared to fish injected with *Fus* AMO alone (i), *Fus* AMO and WT *FUS* (ii), *Fus* AMO and R521H *FUS* (iii), *Fus* AMO and R521C *FUS* (iv) and *Fus* AMO and S57Δ *FUS* (v), or with R521H *FUS* (vi), R521C *FUS* (vii), S57Δ *FUS* (viii) or with WT *FUS* mRNA alone (ix). Arrowheads represent length to the first axonal branching (UAL). Scale bar: 40 µm.

*Fus* KD by AMO caused motor deficits consisting of a significantly abnormal motor behaviour measured as a deficient touch-evoked escape response (TEER) (Figure 2Ai) as well as reduced outgrowth of hyperbranched axons from motor neurons or unbranched axonal length (UAL) (Figure 2Bi) that we subsequently refer to as the motor phenotype. The TEER was deficient in 57% of fish injected but was negligible in zebrafish non-injected (2%), sham injected (3%) or injected with mismatch AMO (7%). Only 26% of larvae injected with *Fus* AMO displayed a normal touch-evoked escape response (Figure 2Ci) as compared to 95% in non-injected, 87% in sham-injected and 82% in larvae injected with the mismatch AMO. Similarly, the length of the motor axons to the point of the first branch (UAL) was significantly reduced in larvae injected with *Fus* AMO when compared to non-injected zebrafish from 99 to 76 µm (Figure 2B, 2Ci). These results are summarized in Table 1 row 1–4.

**Table 1:** Summary of the motor phenotype quantification in the ALS matrix.

Conditions	% Phenotype	% Normal	UAL	N	n	Figure
**Non Injected**	2	94.8	99.2	753	32	n/a
**Mis ** ***Fus*** ** AMO**	6.9	81.6	N.D.	87	3	n/a
**Sham Injected**	3.5	87.7	N.D.	114	4	n/a
***Fus*** ** AMO**	56.9	25.9	75.9	437	12	**2Ci + 3Bi + 5Aiv**
***Fus*** ** AMO + WT ** ***FUS***	16.2	70.3	91.1	185	6	**2Cii+3Bii**
***Fus*** ** AMO + R521H ** ***FUS***	61.9	21.1	74.1	147	4	**2Ciii**
**Fus AMO + R521C ** ***FUS***	34.3	47.9	80.2	169	5	**2Civ**
***Fus*** ** AMO + S57del ** ***FUS***	19.1	63.8	92	94	3	**2Cv**
**R521H ** ***FUS***	38.4	42.6	78.3	284	9	**2Cvi + 3Di + 5Ai**
**R521C ** ***FUS***	18.5	63.1	93.1	157	5	**2Cvii**
**S57del ** ***FUS***	12.2	72.5	97.8	92	3	**2Cviii**
**WT ** ***FUS***	9.8	77.4	93.9	204	6	**2Cix**
***Tardbp*** ** AMO**	58.1	26.6	70.9	327	8	**3Ai + 5Av**
***Tardbp*** ** AMO + WT ** ***TARDBP***	19.9	58.9	86.1	151	4	**3Aiii**
**WT ** ***TARDBP***	18	61	89.1	128	3	n/a
***Tardbp*** ** AMO + WT ** ***FUS***	32.6	49.4	83.4	172	5	**3Aii**
***Fus*** ** AMO + WT ** ***TARDBP***	58.1	27.7	77.1	148	5	**3Biii**
***Fus *** **+ Tardbp AMOs**	59	20.9	69.3	139	4	**5Avi**
**G348C** ***TARDBP***	54.3	23.5	75.6	221	6	**3Ci + 5Aii**
**G348C** ***TARDBP *** **+ R521H ** ***FUS***	47.1	25	75.3	104	3	**5Aiii**
**R521H ** ***FUS *** **+ WT ** ***TARDBP***	30.8	40.7	78.1	91	3	**3Diii**
**R521H ** ***FUS *** **+ WT ** ***FUS***	35.6	41.4	78.9	87	3	**3Dii**
**G348C ** ***TARDBP *** **+ WT ** ***TARDBP***	44	21.1	70.5	109	3	**3Ciii**
**G348C ** ***TARDBP *** **+ WT ** ***FUS***	48.9	22.9	73	96	3	**3Cii**
**WT ** ***SOD1***	8	68.2	N.D.	88	3	n/a
***Tardbp*** ** AMO + WT ** ***SOD1***	58.5	25.5	71.8	94	3	**3Aiv**
***Fus*** ** AMO + WT ** ***SOD1***	57.1	29.5	76.2	112	3	**3Biv**
**G348C** ***TARDBP *** **+ WT ** ***SOD1***	53.8	21.5	75.3	93	3	**3Civ**
**R521H ** ***FUS *** **+ WT ** ***SOD1***	38.8	41.2	75.9	85	3	**3Div**
***Sod1*** ** AMO**	12.3	67.9	N.D.	81	3	n/a
***Sod1 *** **+ ** ***Tardbp*** ** AMOs**	55.8	25.2	71.9	104	3	**3Av**
***Sod1 *** **+ ** ***Fus*** ** AMOs**	58.7	25	75.4	92	2	**3Bv**
***Sod1*** ** AMO + G348C** ***TARDBP***	58.4	22.5	75.6	89	3	**3Cv**
***Sod1*** ** AMO + R521H ** ***FUS***	39.2	40.2	78.2	102	3	**3Dv**
**G93A ** ***SOD1***	33.1	44.4	81.4	223	5	**5Bi**
**G93A ** ***SOD1 *** **+ WT ** ***FUS***	34.6	42.1	76.4	107	3	**5Bii**
**G93A** ***SOD1 *** **+ WT ** ***TARDBP***	33	44	80.2	91	3	**5Biii**
**G93A ** ***SOD1 *** **+ R521H ** ***FUS***	58.8	17	67.9	153	5	**5Biv**
**G93A** ***SOD1 *** **+ G348C ** ***TARDBP***	57.2	19.2	68.4	187	5	**5Bv**

Summary of the motor phenotype quantifications (TEER; % and UAL; µm) for all the conditions described in this study. After each experiment (n), embryos (N) were separated into four groups and the percentages were calculated for dead and developmentally deficient (2–20%, data not shown) as well as normally developed fish with delayed (phenotype) or normal swimming following the TEER. Also the length (µm) of the axonal projections (UAL) from motor neurons was assessed for each group. The last row indicates the Figure where these data are presented.

To determine if the human *FUS* and the zebrafish *Fus* genes are functionally similar, we co-injected human WT *FUS* mRNA alongside *Fus* AMO. The motor phenotype was rescued in these sets of injections when compared to injections of *Fus* AMO alone with significant increases of the percentages of zebrafish displaying a normal TEER from 26 to 70% as well as augmentation of the UAL of motor neurons from 76 to 91 µm (Figure 2A, 2B, 2Ci versus 2A, 2B, 2Cii and Table 1 rows 4–5).

Next, we examined whether mutations in the *FUS* gene can cause loss of function and result in a motor phenotype similar to what we previously demonstrated for *TARDBP* mutations [50]. Contrary to WT *FUS* mRNA, co-injection of R521H *FUS* mRNA and *Fus* AMO (Figure 2Ciii) did not rescue the motor phenotype (21% of zebrafish with normal TEER and the UAL from motor neurons measured at 74 µm). Co-injection of the R521C *FUS* mRNA partially rescued this phenotype, from 26% with AMO (see above) to 48% of zebrafish displaying a normal TEER and UAL of 80 µm (Figure 2Civ and Figure S1A, S1Biii) since it was also found to be significantly different from the rescue of *Fus* KD by WT *FUS* mRNA injection with 70% of fish with normal TEER and UAL of 91 µm (Figure 2A, 2B, 2Cii). However, co-injection of S57Δ *FUS* mRNA completely rescued the phenotype induced by *Fus* AMO (Figure 2Cv and Figure S1A, S1Biv) to a similar degree as the co-injection WT *FUS* mRNA injection (Figure 2A, 2B, 2Cii) with 64% versus 70% of zebrafish displaying a normal TEER and UAL at 92 versus 91 µm. These results are summarized in Table 1 rows 4–8.

### Toxic gain of function of mutant *FUS*

Furthermore, we determined whether expression of WT and ALS-related mutant *FUS* mRNAs by themselves caused a motor deficit in zebrafish. Upon overexpression of R521H *FUS* mRNA in zebrafish embryos we observed a motor phenotype with only 43% of zebrafish larvae displaying a normal TEER (Figure 2Aiv, 2Cvi) as compared to 77% upon WT *FUS* injection (Figure 2Aiii, 2Cix). Similarly, the UAL of motor neurons overexpressing R521H *FUS* (Figure 2Biv, 2Cvi) was reduced to 78 µm as compared to 94 µm upon WT *FUS* injection (Figure 2Biii, 2Cix). Surprisingly, expression at similar levels of the R521C *FUS* mRNA (Figure S1A, S1Bi and Figure 2Cvii) and the S57Δ *FUS* mutations (Figure S1A, S1Bii and Figure 2Cviii) did not elicit a motor phenotype with similar percentages of embryos displaying a normal TEER (R521C: 63% and S57Δ: 72% versus WT: 77%; Figure 2Cvii,viii and Figure S1Ai,ii) as described above for WT *FUS* mRNA (Figure 2Aiii, 2Cix). Similarly, the length of the UAL of motor neurons was not significantly altered when compared to WT *FUS* (Figure 2Cix) (R521C: 93 µm and S57Δ: 98 µm versus WT: 91 µm; Figure 2Cvii,viii and Figure S1Bi,ii). These and subsequent sets of injections presented below did not significantly affect percentages of developmentally deficient (∼10%) and of dead embryos (∼10%) within our conditions (data not shown). The differences observed upon mRNA injections were not due to different levels of protein expression, since Western blot analysis revealed no changes in expression between the WT protein and the three ALS-related FUS mutants described here (Figure 1D). The results from this section are summarized in Table 1 rows 9–12.These data indicate that the ALS-related R521H mutation of *FUS* can cause motor neuron deficits leading to a toxic gain of function.

These *in vivo* results suggest that both toxic gain and loss of function (R521H) or solely loss of function (R521C) can render *FUS* mutations pathogenic. A lack of phenotype for the S57Δ *FUS* could indicate that this variant may be a rare polymorphism not causing disease. In accordance with this functional characterization performed in our zebrafish model, the R521H and R521C *FUS* mutations have been identified in a large number of FALS and SALS cases with both these mutations segregating with disease in large families, whereas the S57Δ variant was identified only in one SALS case [11], [13], [14], [17]–[28].

### Genetic interactions between *FUS* and *TARDBP*

**Figure 3:**
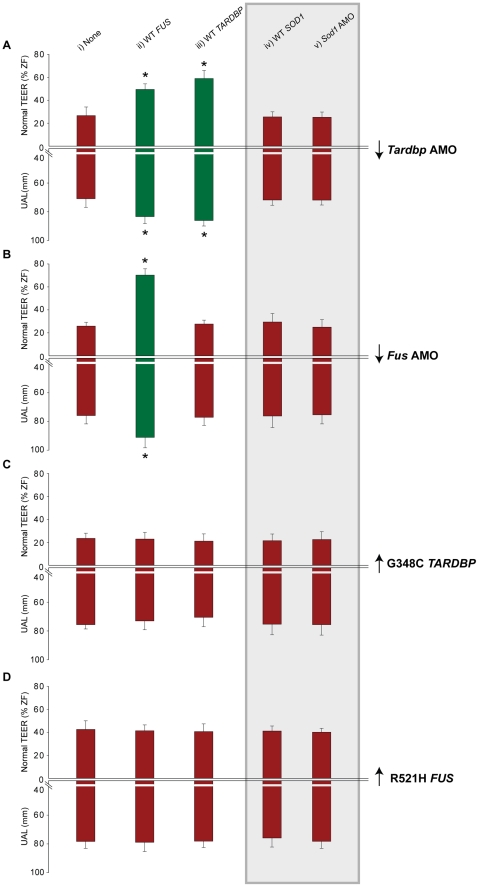
*FUS* and *TARDBP* genetically interact independently of *SOD1*. Percentage of embryos able to swim away having a normal TEER (upward bars, averages of % of normal ZF embryos ± SEM) and the length of primary motor axons (downward bars, averages of UAL in µm ± SEM) were measured upon injection of either A) *Tardbp* AMO (downward scale represents gene knock-down), B) *Fus* AMO, C) G348C mutant *TARDBP* mRNA (upward scale represents mRNA overexpression) or D) R521H mutant *FUS* mRNA alone (none; i) or along with injection of either WT *FUS* mRNA (ii), WT *TARDBP* mRNA (iii), *Sod1* AMO (iv, grey background) or *SOD1* mRNA (v, grey background). Red bars represent failure to rescue the motor phenotype from the KD (A,Bi) or mutant overexpression (C,Di), whereas green bars show rescue (* p<0.05; all values given in Table 1).

**Figure 4:**
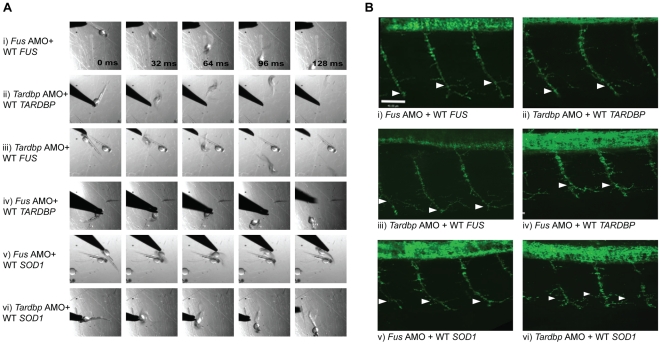
*FUS* rescues the motor phenotype induced by *Tardbp* KD, but not *vice-versa*. A) Motor phenotype was assessed both by time frames obtained from video recordings of the TEER as well as B) immunohistochemical labeling of the axonal projections to determine UAL in motor neurons. The analysis shows that both expression of WT *TARDBP* and WT *FUS* can rescue the motor phenotype caused by KD of *Tardbp* (i) and *Fus* (ii) respectively. Furthermore, WT *FUS* is able to rescue the motor phenotype induced by KD of *Tardbp* (iii), but WT *TARDBP* does not rescue the motor phenotype caused by KD of *Fus* (iv). Similarly, WT *SOD1* is unable to rescue *Fus* (v) and *Tardbp* (vi) KD phenotypes. Arrowheads represent length to the first axonal branching (UAL). Scale bar: 40 µm.

We hypothesized that *FUS* and *TARDBP* operate through a common genetic pathway. To examine this possibility, we tested whether *FUS* or *TARDBP* could rescue the loss of function phenotype caused by knockdown of either of these genes in zebrafish. WT *FUS* mRNA was able to rescue the motor phenotype (49% of zebrafish with normal TEER and UAL of 83 µm) (Figure 3Aii and Figure 4A, 4Biii) caused by *Tardbp* KD alone (27% of zebrafish with normal TEER and UAL of 71 µm) (Figure 3Ai), similarly to the rescue observed with WT *TARDBP* (59% of zebrafish with normal TEER and UAL of 86 µm) (Figure 3Aiii and Figure 4A, 4Bii). In contrast, co-injection of WT *TARDBP* mRNA with *Fus* AMO alone (28% of zebrafish with normal TEER and UAL of 77 µm) (Figure 3Biii and Figure 4A, 4Biv) was unable to rescue the phenotype obtained by KD of *Fus* alone (26% of zebrafish with normal TEER and UAL of 76 µm) (Figure 2A, 2Bi and Figure 3Bi). As mentioned previously, co-injection of WT *FUS* mRNA with *Fus* AMO (Figure 2A, 2Bii and Figure 3Bii) was able to properly rescue the motor phenotype caused by *Fus* KD alone (Figure 2A, 2Bi and Figure 3Bi). The results from this section are summarized in Table 1 row 4, 13–14 and 16–17. These results demonstrate that a genetic interaction between *FUS* and *TARDBP* exists, with *FUS* overexpression being able to rescue the *Tardbp* KD phenotype as a downstream effector.

*TAF15* has a high structural and functional homology to *FUS*, since both TAF15 and FUS belong to the class of TET family of multifunctional DNA/RNA-binding proteins [56]. *TAF15* has been suggested by one study as a candidate gene in ALS with few variants identified solely in FALS cases, but not in controls [57]. We thus determined the motor phenotype upon *Tardbp* KD with or without overexpression of *TAF15* mRNA. Overexpression of *TAF15* mRNA did not cause an overt motor phenotype with 65% displaying a normal TEER. Co-injecting *TAF15* mRNA with *Tardbp* AMO (33% of zebrafish with normal TEER) failed to rescue the motor phenotype caused by *Tardbp* KD alone (27% of zebrafish with normal TEER). (Figure S2). These results further ascertain that the rescue of the phenotype induced by *Tardbp* KD by overexpressing WT *FUS* mRNA is specific to this gene and could be independent of the known functions of FUS and its homologue TAF15, such as DNA and/or RNA binding.

Conversely, we also determined whether the motor phenotype caused by the toxic gain of function of *FUS* could be rescued by overexpression of WT *TARDBP* and *vice-versa* (a dozen combinations; Figure 3A–3D, i–iii), expecting that a toxic gain of function may be irreversible. We and others have previously demonstrated that overexpression of mutant *TARDBP* causes a similar motor phenotype to the one described above, most pronounced upon injection of the G348C mutation [48], [50]. Overexpression of this mutant caused deficits in TEER in 54% of zebrafish with only 23% displaying a normal TEER and reduced UAL of motor axons (76 µm) (Figure 3Ci). Our results show that co-expression of mutant *TARDBP* with either WT *FUS* (23% of zebrafish with normal TEER and UAL of 73 µm) (Figure 3Cii) or WT *TARDBP* (21% of zebrafish with normal TEER and UAL of 70 µm) (Figure 3Ciii) failed to rescue the toxic gain of function phenotype caused by mutant *TARDBP* alone (23% of zebrafish with normal TEER and UAL of 76 µm) (Figure 3Ci). Similarly, we were unable to rescue the motor phenotype caused by expression of the R521H mutant *FUS* (43% of zebrafish with normal TEER and UAL of 78 µm) (Figure 2A, 2Biv and Figure 3Di) with either WT *TARDBP* (41% of zebrafish with normal TEER and UAL of 78 µm) (Figure 3Diii) or WT *FUS* (41% of zebrafish with normal TEER and UAL of 79 µm) (Figure 3Dii). The data from this section are summarized in Table 1 rows 9, 19–24.Thus, overexpression of WT *FUS* or *TARDBP* did not rescue the motor phenotype generated by ALS-related mutations in these two genes, consistent with their toxic gain of function.

**Figure 5:**
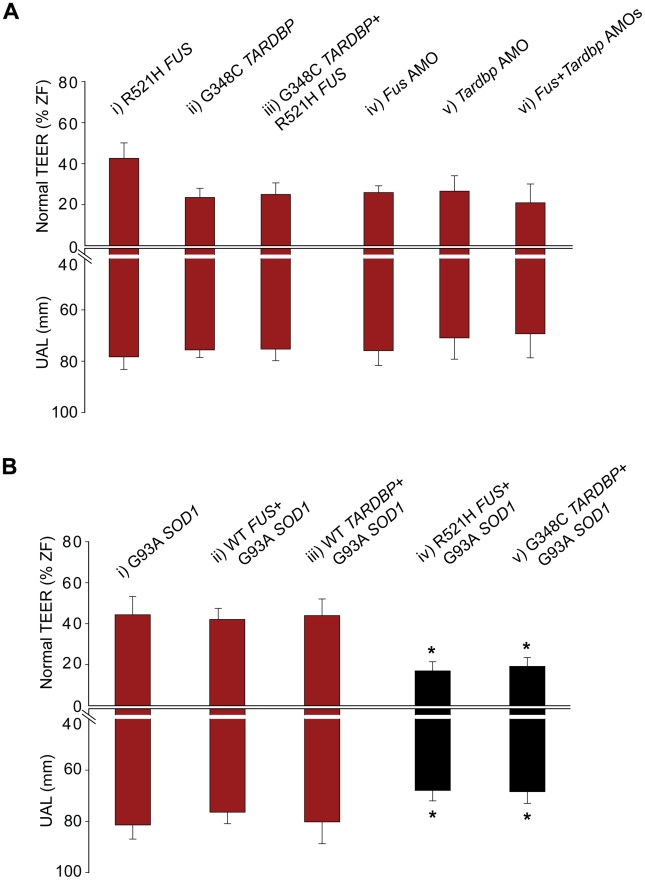
Double mutant and double KD interactions between *SOD1* and *TARDBP/FUS*. A) Percentage for zebrafish with a normal TEER (upward bars, averages of % of normal ZF embryos ± SEM) and the length of the primary motor axons (UAL; downward bars, averages of UAL in µm ± SEM) were measured. Motor phenotypes were observed upon overexpression of mutant R521H *FUS* alone (i), mutant G348C *TARDBP* alone (ii), mutants R521H *FUS* with G348C *TARDBP* (iii), injection of *Fus* AMO alone (iv), *Tardbp* AMO alone (v; as in Figure 2Ai), and co-injection of *Fus* and *Tardbp* AMOs (vi). B) Similarly, motor phenotypes were also observed upon expression of mutant G93A *SOD1* alone (i) as well as co-expression with WT *FUS* (ii), WT *TARDBP* (iii), mutant R521H *FUS* (iv), or mutant G348C *TARDBP* (v). Red bars indicate a significant (p<0.05) motor phenotype as compared to expression of WT *FUS* alone (Figure 2Cix); black bars indicate an exacerbated phenotype compared to Bi (* p<0.05) compared to that observed upon expression of mutant G93A *SOD1* alone (i).

**Figure 6:**
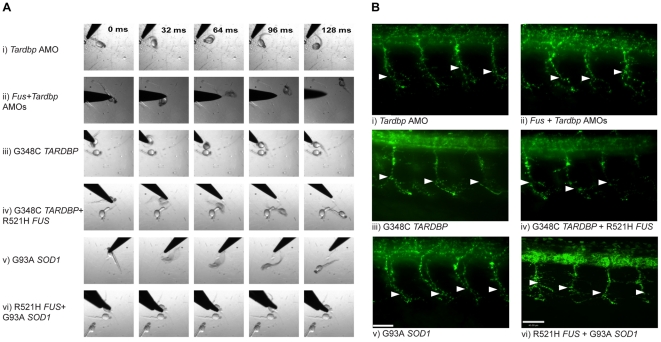
Mutant *SOD1* exacerbates the motor phenotype caused by mutant *FUS* and mutant *TARDBP*. A) Motor phenotype was assessed both by time frames obtained from video recordings of the TEER as well as B) immunohistochemical labeling of the axonal projections to determine UAL in motor neurons. The analysis demonstrates that knock-down of both *Tardbp* and *Fus* (ii) does not lead to an exacerbated phenotype when compared to phenotypes generated by KD of *Tardbp* (i) or *Fus* by AMOs. Similarly, overexpression of both mutant R521H *FUS* and G348C *TARDBP* (iv) does not aggravate the phenotype observed by overexpression of mutant *TARDBP* (iii) or mutant *FUS* (Figure 2A, 2Biv). However, co-expression of mutant *SOD1* and mutant *FUS* did exacerbate the motor phenotype caused by mutant *SOD1* alone (v). Arrowheads represent length to the first axonal branching (UAL). Scale bar: 40 µm.

If *TARDBP* and *FUS* interact, then overexpression of both mutant *TARDBP* and *FUS* mRNAs or simultaneous KD of both genes should yield a similar motor phenotype, whereas an exaggerated, additive phenotype could be expected if these genes act in independent pathways (see below for *SOD1*). As predicted for interacting genes, a similar, non-exacerbated motor phenotype was observed upon injection of the R521H mutant *FUS* (43% of zebrafish with normal TEER and UAL of 78 µm) (Figure 2A, 2Biv and Figure 5Ai), G348C mutant *TARDBP* (23% of zebrafish with normal TEER and UAL of 76 µm), (Figure 5Aii and Figure 6A, 6Biii) or both mutant *TARDBP* and *FUS* (25% of zebrafish with normal TEER and UAL of 75 µm) (Figure 5Aiii and Figure 6A, 6Biv). We also determined that co-injection of *Fus* and *Tardbp* AMOs (21% of zebrafish with normal TEER and UAL of 69 µm) (Figure 5Avi and Figure 6A, 6Bii) did not exacerbate the KD motor phenotype observed upon injection of either *Fus* AMO (26% of zebrafish with normal TEER and UAL of 76 µm) (Figure 5Aiv and Figure 2A, 2Bi) or *Tardbp* AMO (27% of zebrafish with normal TEER and UAL of 71 µm) (Figure 5Av and Figure 6A, 6Bi). These data are summarized in Table 1 rows 4, 9, 13 and 18–20. Since injections of two AMOs might harbor robust effects that would not allow the proper visualization of a possible exacerbation of the motor phenotype, we also injected suboptimal doses (see Materials and Methods) of both *Fus* and *Tardbp* AMOs and compared the motor phenotype with the one induced by single injection of *Fus* and/or *Tardbp* AMOs. The motor phenotype was not significantly altered when subdoses (half) of both *Fus* and *Tardbp* AMOs (61% of zebrafish with normal TEER versus 21% at the higher dose) was compared to single AMO injection of either *Tardbp* (63% of zebrafish with normal TEER versus 26% at the higher dose) and/or *Fus* (69% of zebrafish with normal TEER versus 27% at the higher dose) (Figure S3). Similar results were thus obtained when using either full or subdoses of both *Fus* and *Tardbp* AMOs.

### Lack of genetic interactions between *FUS*/*TARDBP* and *SOD1*

Having provided evidence for an *in vivo* genetic interaction between *TARDBP* and *FUS* we sought to determine whether *SOD1* interacts with these genes by performing further gain and loss of function genetic manipulations using a specific *Sod1* AMO, as well as WT or mutant (G93A) *SOD1* mRNAs. We then sought to determine whether *SOD1* acted downstream, upstream or independently of *TARDBP* or *FUS*, comprising nineteen conditions (Figure 3A–3D iv–v grey background and Figure 4A, 4Bv and Figure 4vi). We first tested if *SOD1* acts downstream of *TARDBP* and *FUS* by examining whether WT *SOD1* could rescue the motor phenotypes generated by loss or toxic gain of function of *TARDBP* or *FUS*. Overexpression of WT *SOD1* did not yield a motor phenotype on its own (motor phenotype consisting of 68% of zebrafish with normal TEER) and failed to rescue the motor phenotype induced by KD of *Tardbp* (25% of zebrafish with normal TEER and UAL of 72 µm) (Figure 3Aiv and Figure 4A, 4Bvi), KD of *Fus*, (29% of zebrafish with normal TEER and UAL of 76 µm) (Figure 3Biv and Figure 4A, 4Bv) as well as overexpression of the G348C mutant *TARDBP* (21% of zebrafish with normal TEER and UAL of 75 µm) (Figure 3Civ), or the R521H mutant *FUS* (41% of zebrafish with normal TEER and UAL of 76 µm) (Figure 3Div). Next, we tested whether KD of *Sod1* could alleviate the motor phenotype caused by mutant *TARDBP* or *FUS*. Injection of an AMO to specifically KD *Sod1* did not cause a motor phenotype on its own (consisting of 68% of zebrafish with normal TEER), consistent with the lack of phenotype observed in *SOD1* knockout mice [58]. In contrast, co-injection of AMOs to *Sod1* and *Tardbp* (25% of zebrafish with normal TEER and UAL of 72 µm) (Figure 3Av) or to *Sod1* and *Fus* (25% of zebrafish with normal TEER and UAL of 75 µm) (Figure 3Bv) yielded a similar motor phenotype to that observed upon KD of *Tardbp* (motor phenotype consisting of 27% of zebrafish with normal TEER and UAL of 71 µm) (Figure 3Ai and Figure 6A, 6Bi) or *Fus* (26% of zebrafish with normal TEER and UAL of 76 µm) (Figure 2A, 2Bi and Figure 3Bi) alone. Co-injection of *Sod1* AMO with mutant *TARDBP* (22% of zebrafish with normal TEER and UAL of 75 µm) (Figure 3Cv) or mutant *FUS* (40% of zebrafish with normal TEER and UAL of 78 µm) (Figure 3Dv) also failed to modify the motor phenotype obtained by injecting mutant *TARDBP* alone (21% of zebrafish with normal TEER and UAL of 75 µm) (Figure 3Ci and Figure 6A, 6Biii) and mutant *FUS* alone (motor phenotype consisting of 43% of zebrafish with normal TEER and UAL of 78 µm) (Figure 2A, 2Biv, Figure 3Di). From these combined results we can infer that *SOD1* is not acting downstream of *TARDBP* or *FUS*. These results are summarized in Table 1 rows 4, 9, 13, 19 and 25–34.

Alternatively, to determine if *SOD1* acts upstream we tested for rescue of the mutant *SOD1*-induced motor phenotype by overexpressing WT *TARDBP* or *FUS*. Overexpression of mutant *SOD1* mRNA in zebrafish embryos has been shown to cause shortening and premature branching of axonal projections from the motor neurons in the spinal cord [8] and is consistent with the toxic gain of function observed in ALS [2], [7], [8]. Consistent with these published results, we observed a motor phenotype consisting of 33% of zebrafish with swimming deficits, 44% of zebrafish with normal TEER and UAL of 81 µm when we overexpressed the G93A mutant SOD1 in our zebrafish model (Figure 5Bi and Figure 6A, 6Bv). Similarly, co-expression with mutant *SOD1* of WT *TARDBP* (motor phenotype consisting of 44% of zebrafish with normal TEER and UAL of 80 µm) (Figure 5Biii) or *FUS* (42% of zebrafish with normal TEER and UAL of 76 µm) (Figure 5Bii) failed to rescue this motor deficit to any significant extent, indicating that mutant *SOD1* does not act upstream of either of these two genes. These results are summarized in Table 1 rows 9, 19 and 35–37.

### *SOD1* acts independently of *FUS*/*TARDBP*

The foregoing results strongly suggest that *SOD1* acts independently of *TARDBP* and *FUS* in our models. If this is true, then the motor phenotype yielded by either mutant *SOD1* or *TARDBP/FUS* alone should be less severe than the “additive” phenotype of mutant *SOD1* and *TARDBP/FUS*. Indeed, co-injection of both the R521H mutant *FUS* and the G93A mutant *SOD1* mRNAs (motor phenotype consisting of 17% of zebrafish with normal TEER and UAL of 68 µm) (Figure 5Biv and Figure 6A, 6Bvi) yielded an exaggerated motor phenotype with a higher percentage of embryos affected as well as an exacerbated axonal shortening from motor neurons when compared to injection of mutant *FUS* alone (42% of zebrafish with normal TEER and UAL of 78 µm) (Figure 2A, 2Biv and Figure 5Ai) or mutant *SOD1* (44% of zebrafish with normal TEER and UAL of 81 µm) (Figure 5Bi and Figure 6A, 6Bv) alone. Similarly, co-injection of mutant *SOD1* and of mutant *TARDBP* (motor phenotype consisting of 19% of zebrafish with normal TEER and UAL of 68 µm) (Figure 5Bv) led to a significantly exacerbated motor phenotype when compared to mutant *SOD1* alone (44% of zebrafish with normal TEER and UAL of 81 µm) (Figure 5Bi and Figure 6A, 6Bv). The data in this section are summarized in Table 1 rows 9, 19 and 38–39. These results indicate that mutant *SOD1* may yield a motor phenotype which is additive with that generated by ALS-related *FUS* and *TARDBP* mutations, suggesting that *SOD1* may act independently of *TARDBP* and *FUS*.

## Discussion

### Does a central pathogenic mechanism involving genetic interactions of *FUS* and *TARDBP* lead to motor neuron degeneration in ALS?

**Figure 7:**
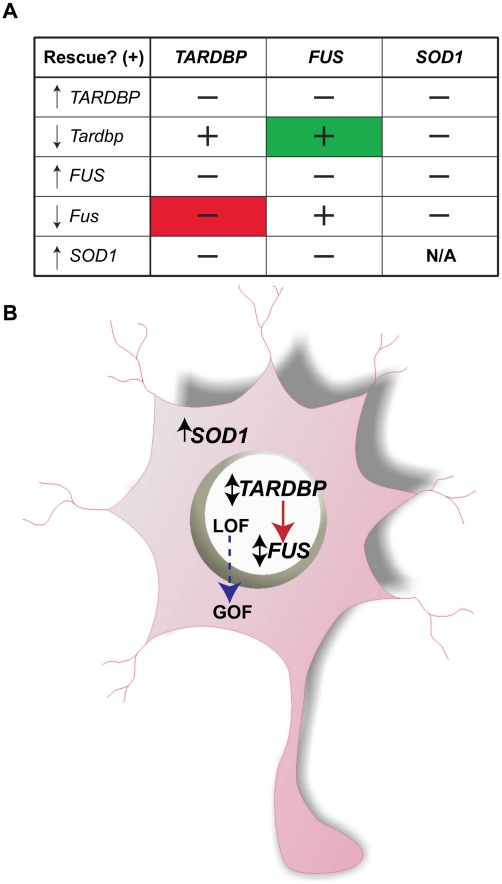
Summary of multigenic interactions and a hypothetical ALS molecular network. A) A genetic matrix of the main interactions described here with ↑ representing phenotype due to overexpression of mutant mRNA and ↓ representing phenotype due to KD of the zebrafish mRNA and ↕ for both phenotypes. ‘−’ stands for lack of rescue (i.e. motor phenotype), ‘+’ represents rescue. Note in green the rescue of the *Tardbp* KD phenotype by WT *FUS* mRNA and in red the lack of rescue of the *Fus* KD phenotype by WT *TARDBP* mRNA. B) Cartoon summary of the proposed ALS network with ↑ representing motor phenotype due to overexpression of mutant mRNA and ↓ representing phenotype due to KD of the zebrafish mRNA and ↕ for both phenotypes in which nuclear expression of *TARDBP* is upstream of *FUS* and both are independent of cytosolic expression of *SOD1*. Loss of function (LOF) for *FUS*/*TARDBP* could be due to mislocalization from the nucleus, whereas gain of function (GOF) due to abnormal levels of these aggregate-prone cytosolic proteins.

As summarized in the matrix of Figure 7A, here we show that expression of WT *FUS* is able to rescue the motor phenotype induced by KD of *Fus* (Figure 2) as well as the motor phenotype caused by KD of zebrafish *Tardbp* (Figure 3 and Figure 4; summarized in Figure 7A, green cell). On the other hand, expression of WT *TARDBP* is unable to rescue the phenotype caused by KD of *Fus* (Figure 3 and Figure 4; Figure 7A, red cell), whereas it does rescue the motor phenotype induced by KD of *Tardbp*. These results as well as *in vitro* reports of physical interactions [35], [36] suggest that *TARDBP* and *FUS* share a common genetic pathway, with *FUS* being downstream of *TARDBP*. Alternatively, as certain studies have previously demonstrated, *FUS* could be a more general transcriptional regulator, potentially capable of compensating for certain of the functions of *TARDBP* [15]. This possibility is less likely since overexpression of *TAF15*, a gene belonging to the class of TET family of multifunctional DNA/RNA-binding proteins with high functional and structural similarity to *FUS* was unable to rescue the motor phenotype caused by *Tardbp* KD (Figure S2). The lack of exacerbation of partial or more complete double knockdowns also indicates a non-additive effect of *TARDBP* and *FUS* (Figure 5 and Figure S3). Although we were unable to generate an increased phenotype upon double KD of *Tardbp* and *Fus* in this study, we cannot exclude the possibility of phenotype exacerbation at different doses of AMOs.

Expression of *SOD1* was unable to rescue the phenotypes caused by KD of *Fus* and/or *Tardbp* suggesting independent molecular pathways (Figure 5 and Figure 6). Further, mutant *SOD1* does not appear to genetically converge with the pathogenic cascades elicited by mutant *TARDBP* or *FUS*, since expression of mutant *SOD1* exacerbates the motor phenotype induced by mutant *FUS* (as summarized in Figure 7A).

Although the pathological mechanisms through which ALS-related *TARDBP* mutations cause motor neuron degeneration are not understood, protein misfolding and phosphorylation, nuclear to cytosolic shuttling and RNA imbalance are presumed to be involved [11], [15], [16]. Our results suggest that certain ALS-related *FUS* mutations, similarly to *TARDBP* [50], can cause motor neuron deficits through both loss and toxic gain of function mechanisms. As illustrated in Figure 7B, the molecular mechanisms that cause motor neuron degeneration could be initiated with a loss of function of *FUS* or *TARDBP* due to mislocalization from the nucleus to the cytosol, whereas the toxic gain of function could be the result of abnormal accumulation of aggregate-prone proteins as reported for two of the *FUS* mutations described here, R521H and R521C [14], [54], [55], as well as the mutations R495X, R522G and P525L [59]–[61]. A similar toxic gain of function has been described for the A315T, G348C and A382T *TARDBP* mutations [48], [50]. In fact, several of these recent studies and others have shown that in neurons, expression of TDP-43 and/or FUS in the cytosol causes aggregation of these proteins with subsequent recruitment into stress granules, thus initiating pathogenic events [59]–[63].

While this article was under review, a study in *Drosophila* performed a functional characterization of several *FUS* mutations, including the R521H and R521C mutations described here [37]. When strongly overexpressed solely in neurons mutant *FUS* led to an increased severity of the “rough-eye” phenotype, widespread neurodegeneration and lethality as compared to WT *FUS* overexpression [37]. The authors also suggested genetic interactions between *TARDBP* and *FUS* since overexpression of both mutants together caused exacerbation of this phenotype as compared to expression of either mutant *TARDBP* or *FUS*. However, overexpression of WT *TARDBP* and WT *FUS* together caused similar increases in phenotype severity when compared to overexpression of either WT genes alone [37]. Thus, this exacerbation could be likely a result of excessive amounts of these proteins, which have been found as likely to aggregate in a number of models, including yeast [64]–[66]. On the other hand, we describe here a genetic interaction due to rescue (as opposed to exacerbation) by WT *FUS* of the motor phenotype upon *Tardbp* KD, suggesting that *FUS* is downstream to *TARDBP* in this pathway.

Consistent with a possible action downstream from *TARDBP*, *FUS* is thought to have a more critical role in regulating neuronal morphology and connectivity [15]. In cell lines, TDP-43 was shown to form complexes with hnRNPs and a fraction of TDP-43 in these complexes does interact directly with FUS, with this *in vitro* interaction being enhanced in cell lines from ALS patients harboring *TARDBP* mutations [35]. Another study using cell lines demonstrated that a common biochemical pathway exists where FUS and TDP-43 interact by binding competitively *HDAC6* mRNA, with TDP-43 being upstream in this pathway. Further, since FUS antibodies have been shown to co-label TDP-43 positive protein aggregates observed in both SALS and FALS cases, a similar pathogenic function for these mutant proteins has been suggested [33]. Finally, ubiquitinated aggregates observed in FALS cases with *SOD1* mutations were not immunopositive against TDP-43 or FUS antibodies [33], [34], again consistent with independent pathogenic mechanisms for SOD1 and TDP-43/FUS.

### Interactors of *TARDBP* are central in ALS genetics and pathology

Interestingly, a number of *in vivo* studies have demonstrated that *TARDBP* has a number of genetic interactors such as Ataxin-2 [46], progranulin (*GRN*) [48], [67]–[69], valosin-containing protein (*VCP*) [70], [71] and histone deacetylase 6 (*HDAC6*) [36], [38]. Most of these interactors are mutated in other neurodegenerative disorders, such as dementia and expanded polyglutamine repeat disorders, whereas some may participate in generalized processes such as autophagy. Further, recent genetic studies have shown that mutations in Ataxin-2 and *VCP* are prevalent in ALS patients, with intermediate polyglutamine expansions significantly associated with ALS [46] and a study finding *VCP* mutations in 1–2% of FALS patients [72]. These combined results suggest that *TARDBP* plays a pivotal role in the pathogenic pathways leading to motor neuron degeneration culminating in ALS. These results also suggest that a multigenic pathway shared in a number of neurodegenerative disorders may exist. Unraveling the molecular and genetic components of this network of neurodegenerative interactions could have major implications in our understanding of the pathophysiology of these neurological disorders and could accelerate the discovery of future treatments for these increasingly prevalent diseases.

## Materials and Methods

### Ethics statement

Zebrafish were raised from a colony maintained according to established procedures. All procedures described here were carried out in compliance with the Canadian Council for Animal Care.

### Embryonic RNA manipulation

Injections were performed in 1–4 cell stage blastulae. *FUS* WT and mutants (R521H, R521C, S57Δ), *TARDBP* WT and mutant (G348C), *SOD1* WT and mutant (G93A) mRNAs were transcribed from *NotI*-linearized pCS2+ using SP6 polymerase with the mMESSAGE Machine Kit (Ambion). This was followed by a phenol-chloroform purification and ethanol precipitation, and diluted in nuclease-free water (Ambion). The mRNAs were diluted in nuclease free water (Ambion) with 0.05% Fast Green vital dye (Sigma) at a concentration of 60 ng/µl (*FUS*), 25 ng/µl (*TARDBP*) and 100 ng/µl (*SOD1*) and were pulse-injected into early embryos using a Picospritzer III (General Valve) pressure ejector. The zebrafish *TARDBP, FUS and SOD1* gene orthologues, *Tardbp, Fus, and Sod1* (NM_201476; NM_201083.2; and NM_131294.1 respectively) were identified using the Ensembl's gene homology prediction program (http://www.ensembl.org). AMOs were designed to bind and inhibit specifically the ATG of the following genes (and no other genomic sequence): *Fus* (GGCCATAATCATTTGACGCCATGTT), *Tardbp* (GTACATCTCGGCCATCTTTCCTCAG) and *Sod1* (GCACACAAACGGCCTTGTTCACCAT) mRNA translation (Gene Tools) were designed complimentary to the region of translational initiation of the *Fus* CGAAGGCGACTGTACGTATAACACCTCAGAAATTGTTATTCTGCATCATTTCTAAAAGGATTTTAAGCCCAAAC [(ATG)GCGTCAAATGATTATGGCC]AAA, *Tardbp* (GGAAACAGTTAGCACAGCTCGCGCATTCGGTGTAATC [(ATG)ACGGAGTGCTATATTCGTGTGG]), and *Sod1* TCTTATCAAACACAGTCGGTTTCTTTCACTCTCTCACAACTTCTCAGTTTGCATAATCTACAGTCAGC [(ATG)GTGAACAAGGCCGTTTGTGTGC] mRNAs to inhibit protein translation. These AMOs were designed to bind solely to the 1^st^ ATG of the appropriate region of translational initiation for each gene and were confirmed by BLAST searches to not recognize any other sequences in the zebrafish genome (or any human transcripts). An AMO having nucleotides that were mismatched represented as lowercase to disrupt specificity were also designed for *Fus* (GGCgAaAATgATTTcACcCCATGTT). Dose-dependence curves of AMO and mRNA toxicity were performed and AMOs were injected at a concentration of 0.6 (*Fus*), 0.1 (*Tardbp*) and 0.5 (*Sod1*) mM to minimize morpholino-induced developmental delay and toxicity and to yield a consistent motor phenotype. Suboptimal doses were established for *Fus* (0.3 mM) and *Tardbp* (0.05 mM).

### Touch-evoked escape response (TEER)

Morphology and behavioral touch responses were assessed with a stereomicroscope (Zeiss, Oberkochen, Germany) and only fish with no obvious developmental deficits were selected to determine the TEER. For escape swimming at 48 hpf, embryos were touched lightly at the level of the tail or head with a pair of blunt forceps. Fish that were unable to escape were touched several times (3–4 times) in order to ascertain their failure to respond. Thus, for each injection set, larvae were separated in four groups; dead and developmentally deficient, fish with deficits in TEER and fish displaying a normal TEER. The percentages for the two last groups are described in Table 1 for each condition. Their responses were also recorded using a Photron (San Diego, CA) Fastcam PCI high-speed video camera at a rate of 125 frames/s.

### Immunohistochemistry

For immunohistochemical analysis of axonal projections of motor neurons, monoclonal antibodies anti-SV2 (Developmental Studies Hybridoma) were used to assess the motor neuron morphology at 48 and 72 hpf. Fluorescent images of fixed embryos were taken using a Quorum Technologies spinning-disk confocal microscope mounted on an upright Olympus BX61W1 fluorescence microscope equipped with an Hamamatsu ORCA-ER camera. Image acquisition was performed with Volocity software (PerkinElmer). As previously described [50], axonal projections from primary and secondary motor neurons at a defined location in the intersomitic segments were determined. Analysis of Z-stacks by confocal microscopy was performed in three to four axonal projections per animal. The axonal length to the first branching (UAL) was determined by tracing the labeled axon from the spinal cord to the point where it branches using Image J. These values were averaged for each of the animal analyzed (10–30 zebrafish per condition) for the various conditions in our study.

### *In situ* hybridization

Sense and antisense probes of 500 bp length against *FUS* mRNA were designed. 24 hpf zebrafish embryos were processed for *in situ* hybridization using fluorescent FastRed as previously described [73], with minor modifications.

### Western blotting

Zebrafish embryos were lysed in ice using cold SDS sample buffer (63 mM Tris-HCl pH 6.8, 10% glycerol, 5% ß-mercaptoethanol, 3.5% sodium dodecyl sulfate) and were maintained on ice and homogenized using a hand-held pestle. The lysates were centrifuged for 15 minutes at 13000 rpm and separated into soluble and insoluble fractions. SDS/PAGE Western blotting of both fractions were carried out as previously described [9], using monoclonal antibodies against myc (Invitrogen), actin (Clone C4; ICN BIOMEDICALS, Inc.), a polyclonal antibodies against TDP-43 (ProteinTech) and a polyclonal antibody (Bethyl Laboratories) as well as a monoclonal antibody against FUS (BD Transduction Laboratories).

### Statistics

Statistical significance was determined by anova and post hoc analysis by Tukey's multiple comparison test using Prism software (Prism Software Ltd.) as well as a two-tailed distribution, two-sample equal variance t-test using Sigma Plot software (Systat Software Inc., San Jose, CA, USA). Significance was established at p<0.05.

## Supporting Information

Figure S1 *FUS* mutations are unable to rescue the phenotype induced by KD of *Fus*. A) and B) Motor phenotype was assessed both by time frames obtained from video recordings of the TEER as well as immunohistochemical labeling of the axonal projections of motor neurons to determine the UAL. The analysis demonstrates that two ALS-related mutations, R521C (i) and S57Δ (ii), unlike the R521H mutation, do not induce a significant motor phenotype when compared to WT FUS mRNA expression. Whereas R521C (iii) is unable to rescue the motor phenotype induced by KD of *Fus*, WT (Figure 2) and the S57Δ mutation are able to rescue the motor phenotype caused by *Fus* KD in zebrafish embryos. All quantifications of the motor phenotype are given in Figure 2 and Table 1. Scale bar: 40 µm.
(TIF)

Figure S2 *TAF15*, a *FUS* homologue is unable to rescue the TEER induced by KD of *Tardbp*. A) Representative videos showing the TEER when *TAF15* mRNA was overexpressed (i), upon *Tardbp* KD (ii), and co-injection of the *Tardbp* AMO and *TAF15* mRNA. B) Overexpression of *TAF15* mRNA was unable to rescue the TEER induced by *Tardbp* KD.
(TIF)

Figure S3 KD of both *Tardbp* and *Fus* in zebrafish does not induce an exacerbated motor phenotype. A) Representative videos showing the TEER of zebrafish larvae when subdoses of *Fus* AMO (i), *Tardbp* AMO (ii) and co-injection of both these AMOs (iii). B) An exacerbated TEER phenotype was not observed upon co-injection of both these AMOs.
(TIF)
