# Supplementary figures and images for: FUS and TARDBP but Not SOD1 Interact in Genetic Models of Amyotrophic Lateral Sclerosis
Source: PLoS Genet. 2011 Aug 4;7(8):e1002214. doi: 10.1371/journal.pgen.1002214 (PMC3150442; doi:10.1371/journal.pgen.1002214)

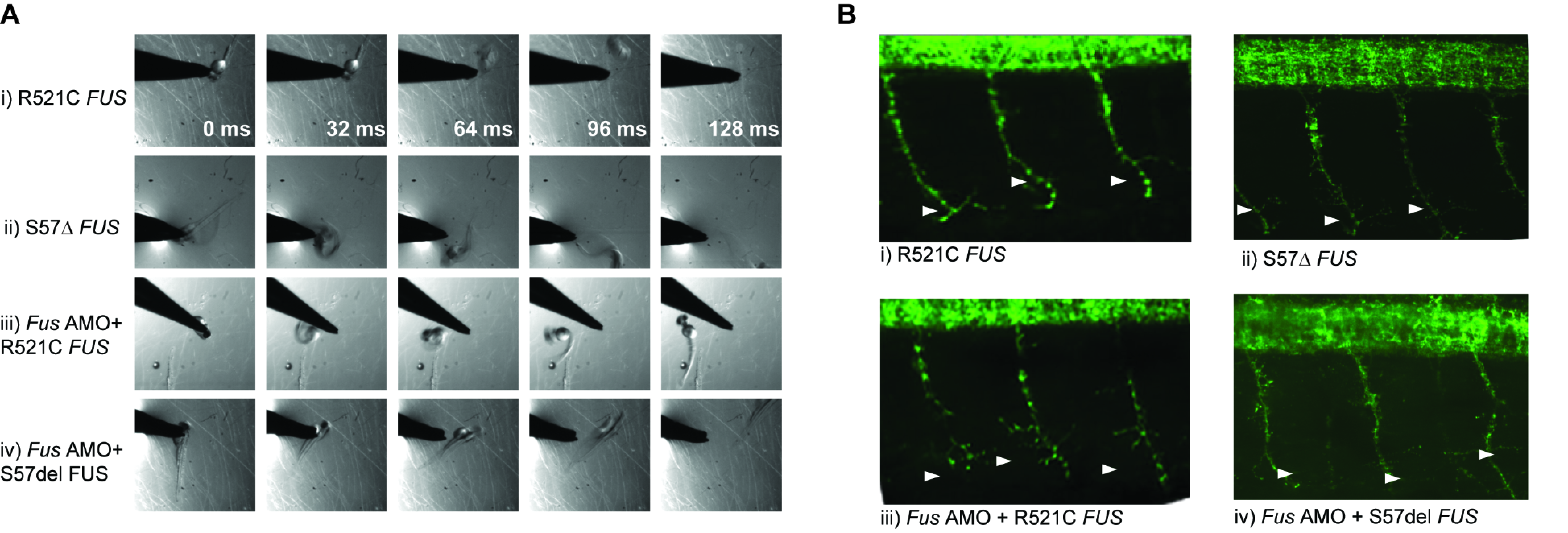

Supplement: Figure S1 — FUS mutations are unable to rescue the phenotype induced by KD of Fus. A) and B) Motor phenotype was assessed both by time frames obtained from video recordings of the TEER as well as immunohistochemical labeling of the axonal projections of motor neurons to determine the UAL. The analysis demonstrates that two ALS-related mutations, R521C (i) and S57Δ (ii), unlike the R521H mutation, do not induce a significant motor phenotype when compared to WT FUS mRNA expression. Whereas R521C (iii) is unable to rescue the motor phenotype induced by KD of Fus, WT (Figure 2) and the S57Δ mutation are able to rescue the motor phenotype caused by Fus KD in zebrafish embryos. All quantifications of the motor phenotype are given in Figure 2 and Table 1. Scale bar: 40 µm. (TIF) [file pgen.1002214.s001.tif]

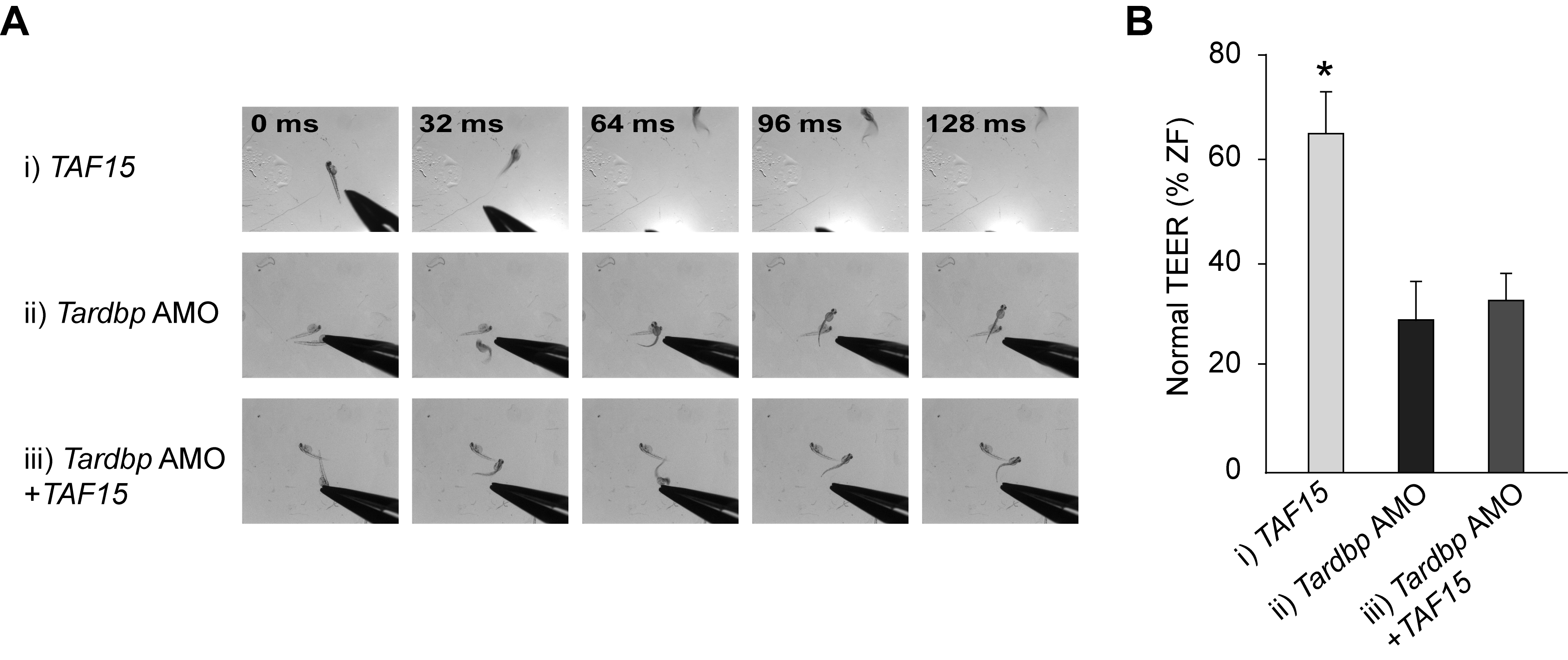

Supplement: Figure S2 — TAF15, a FUS homologue is unable to rescue the TEER induced by KD of Tardbp. A) Representative videos showing the TEER when TAF15 mRNA was overexpressed (i), upon Tardbp KD (ii), and co-injection of the Tardbp AMO and TAF15 mRNA. B) Overexpression of TAF15 mRNA was unable to rescue the TEER induced by Tardbp KD. (TIF) [file pgen.1002214.s002.tif]

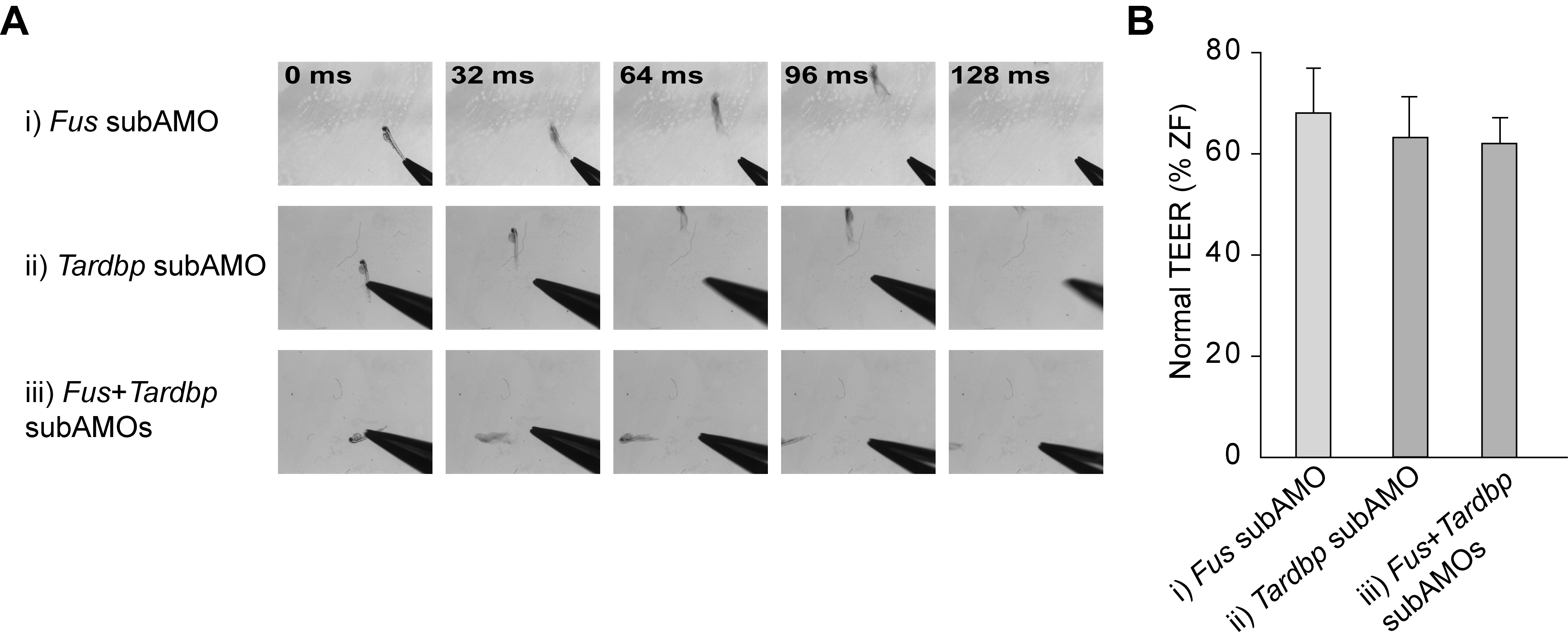

Supplement: Figure S3 — KD of both Tardbp and Fus in zebrafish does not induce an exacerbated motor phenotype. A) Representative videos showing the TEER of zebrafish larvae when subdoses of Fus AMO (i), Tardbp AMO (ii) and co-injection of both these AMOs (iii). B) An exacerbated TEER phenotype was not observed upon co-injection of both these AMOs. (TIF) [file pgen.1002214.s003.tif]
